# Mucosal infection with Tsukamurella species following nasal septum procedure: a rare case report

**DOI:** 10.1097/MS9.0000000000002515

**Published:** 2024-08-30

**Authors:** Alamjeet K. Sidhu, Shreya Khandelwal, Francis V. Dominic Savio, Simranjeet Bedi, Yashash D. Pathak

**Affiliations:** a Kharkiv National Medical University, Ukraine; b B.P. Koirala Institute of Health Sciences, Nepal; c Tianjin Medical University School of Basic Medical Sciences, Heping District, Tianjin, People’s Republic of China; d Hospitalist Baylor College of Medicine, Houston, Texas, USA

**Keywords:** management, pathogens, sinusitis, treatment, Tsukamurella

## Abstract

**Introduction and importance:**

Tsukamurella species are rare, aerobic, gram-positive bacteria known to cause infections, primarily in immunocompromised individuals. This case report presents a rare instance of a mucosal infection caused by Tsukamurella species following a nasal septum procedure in an immunocompetent patient.

**Case presentation:**

A 51-year-old man with a history of multiple hereditary exostosis, allergic rhinitis, and recent nasal fracture repair presented with persistent fevers and low back pain. Postoperatively, he developed sinus pain and small oral lesions, initially treated with antibiotics for presumed sinusitis. Despite treatment, his fever persisted, leading to an emergency department visit. Laboratory tests indicated sepsis, but a CT scan of the sinuses showed no sinusitis. Despite broad-spectrum antibiotics, the patient’s fever continued. On admission day 9, nasal endoscopy and culture identified Tsukamurella species. The patient was treated with augmentin, fluconazole, and levofloxacin, leading to the resolution of symptoms and discharge with ongoing treatment.

**Clinical discussion:**

Tsukamurella species are uncommon pathogens that are often associated with bacteremia in immunocompromised individuals. This case highlights the diagnostic challenges and the importance of considering unusual pathogens in postprocedural infections, even in immunocompetent patients. Accurate identification and appropriate management are critical in improving outcomes for patients with Tsukamurella infections.

**Conclusion:**

This case underscores the need for vigilance in diagnosing rare infections like Tsukamurella, even in immunocompetent individuals. The successful resolution with combination therapy highlights the importance of appropriate antibiotic selection in managing such infections.

## Introduction

Highlights

- A 51-year-old man developed a rare mucosal infection caused by Tsukamurella species following a nasal septum procedure, presenting with prolonged fever and sinus pain.
- Despite treatment for presumed sinusitis, the patient continued to experience fever and sinus pain, leading to an emergency department visit where initial tests indicated a septic condition.
- Thorough evaluation, including bedside nasal endoscopy, culture, and biopsy, was performed to identify the source of infection. Preliminary results suggested a fungal infection, prompting initial antifungal treatment.
- Further culture results revealed Tsukamurella species and Enterococcus faecalis. The patient was treated with augmentin, fluconazole, and levofloxacin, leading to the resolution of the fever.
- This case underscores the importance of considering rare pathogens like Tsukamurella in postsurgical infections and highlights the need for prompt, appropriate management to improve patient outcomes.

Tsukamurella species are obligate aerobic, gram-positive, weak acid-fast, nonmotile bacilli. They are found in various environments, such as soil, water, sludge, and petroleum reservoir wastewater, and belong to actinomycetales. Tsukamurella species are clinically considered to be a rare opportunistic pathogen because most reported cases have been related to bacteremia and intravascular prosthetic devices and immunosuppression^1^. Tsukamurella was first isolated in 1941 from the mycetoma and ovaries of the bedbug. Nine species of the genus Tsukamurella have been isolated from human infections and Tsukamurella commonly causes pneumonia, brain abscesses, catheter-related bloodstream infections, ocular infections, bacteremia, and septic pulmonary emboli in immunocompromised individuals^1,2^.

In rare instances, the Tsukamurella species, predominantly Tsukamurella tyrosinosolvens, and Tsukamurella pulmonis, are responsible for ocular and other mucosal infections, presenting a broader spectrum of ocular manifestations than previously documented^3^. These species are known to cause various ophthalmologic infections, including conjunctivitis, keratitis, blepharitis, and canaliculitis^4^. Tsukamurella exhibits laboratory similarities to mycobacteria and should be considered in the differential of atypical infection^5^. Tsukamurella species are also rare to cause infections in immunocompetent hosts. Human infections with Tsukamurella species are uncommon since these bacteria are typically saprophytes. Consequently, most available information about this species is derived from individual case reports.

Here, we present a rare case of sepsis with persistent bacteremia and frontal sinusitis, which evolved from a mucosal infection in an immunocompetent individual. In accordance with the CARE 2023 guidelines, this case report provides a comprehensive overview of a rare infection caused by Tsukamurella species, ensuring compliance with the latest standards in medical case reporting^6^.

## Presentation

A 51-year-old male with multiple hereditary exostosis, allergic rhinitis, and a recent nasal fracture, for which he underwent nasal repair, presented with low back pain aggravated with physical activity. He was treated symptomatically with Cyclobenzaprine 5 mg. Postoperatively, the patient experienced sinus pain and pressure and a course of amoxicillin and clavulanate was initiated considering sinusitis. Despite the course of antibiotics the patient began to develop fevers. Initially, these were experienced twice daily, but over time, the fevers persisted mainly at night, reaching a maximum temperature of 100.8°F. Under the impression of sepsis due to unknown cause, the patient was admitted and treated empirically with, metronidazole 500 mg, and levofloxacin. The qSOFA score was calculated to be zero at the time of admission.

Table 1 presents the laboratory findings obtained during the patient’s hospital stay, highlighting key indicators of the patient’s septic condition.

**Table 1:** Laboratory findings of the patient

Labs	Day-1	Day-2	Day-4	Day-5	Day-9	Units
WBC	16.8*	17.2*		17.9*	19.4*	K/µl
HGB	12.0*	11.0*		11.6*	11.3*	GM/Dl
HCT	36.7*	33.8*		34.3*	34*	%
PLT	719*	624*		599*	581*	K/CU MM
MCV	83	82		81	83	fl
NA	133*	133*		137	134*	meq/l
K	4.3	4.3		4.0	3.6	meq/l
CL	100	100		106	101	meq/l
Bun	14	14		11	11	mg/dl
Creatinine	0.86	0.86		0.79	0.83	mg/dl
Glucose	106*	106*		113*	140*	mg/dl
Calcium	9.7	9.7		9.0	9.2	mg/dl
Protein	7.5	7.5		6.7	7.1	gm/dl
Bilirubin total	0.3	0.3		0.4	0.8	mg/dl
Alkphos	80	80		60	80	U/l
ALT	12	12		9	16	U/l
AST	12	12		52*	19	U/l
ANA			Positive			
ANA Pattern			Speckled			
ANA Titer			1:40			
Rheumatoid Factor			Negative			

* denotes values that are outside the normal reference range, highlighting the abnormality during patient hospital stay.

Further investigations revealed opacification of the frontal and ethmoid sinuses on the CT sinus, suggestive of sinusitis.

**Figure 1:**
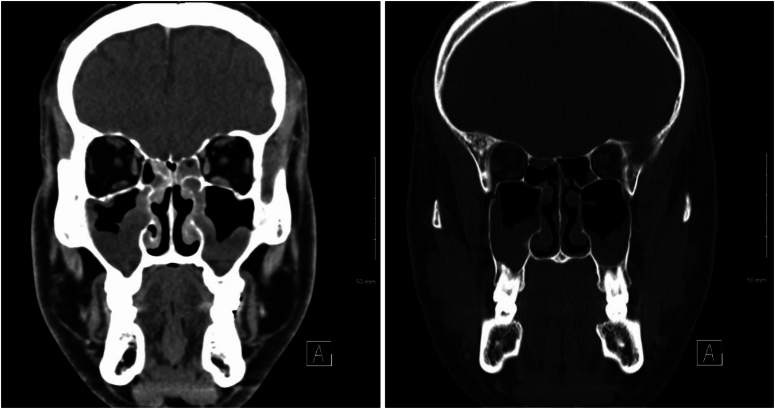
CT maxillofacial with IV contrast: opacification of the frontal and ethmoid sinuses suggestive of sinusitis. Paranasal sinus polypoid soft tissue densities indicate possible sinonasal polyposis or retention cysts.

Further investigations, as depicted in Figure 1, revealed significant opacification of the frontal and ethmoid sinuses on the CT scan of the maxillofacial area with IV contrast. This finding is suggestive of sinusitis, with the scan also revealing polypoid soft tissue densities in the paranasal sinuses, consistent with sinonasal polyposis or retention cysts, which support the diagnosis of sinusitis and guide further management.

**Figure 2:**
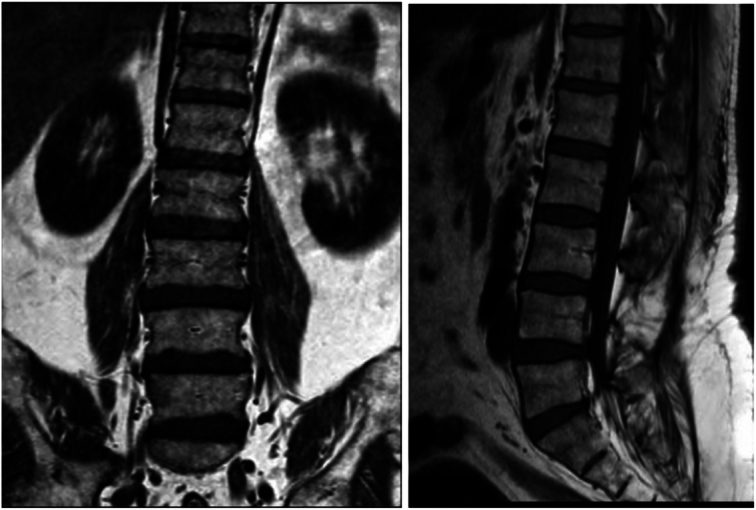
MRI lumbar spine with and without IV contrast: slight enhancement of the cauda equina can be seen along with arachnoiditis.

Figure 2 illustrates the MRI of the lumbar spine, performed with and without IV contrast. The imaging shows slight enhancement of the cauda equina, correlating with the patient’s reported lower back pain and confirming the diagnosis of arachnoiditis.

**Figure 3:**
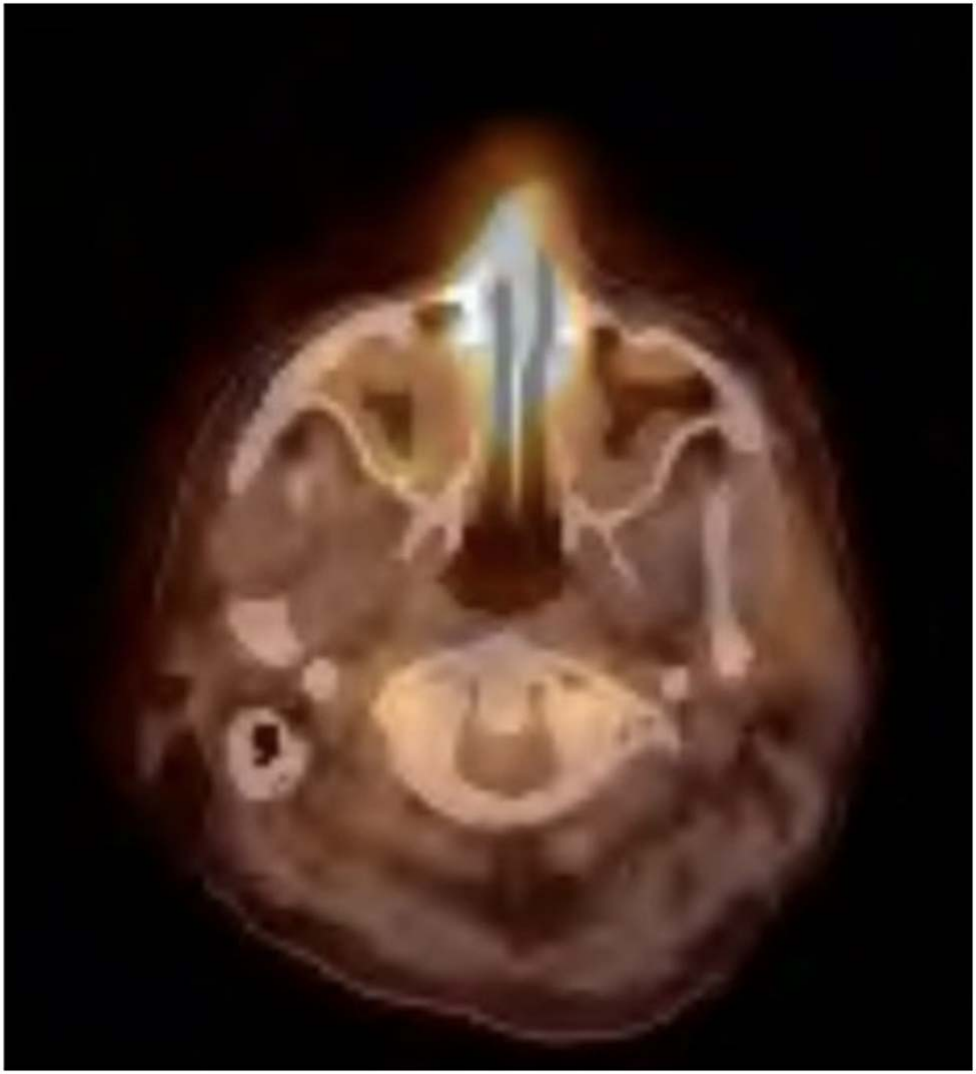
PET-CT of the Skull base: nonspecific moderate increased activity within the nasal cavity in the vicinity of the septum possibly due to inflammation.

Venous duplex imaging showed no evidence of thrombus. The neurologist confirmed the cause of lower back pain was due to arachnoiditis, but was concerned about the septicemia and advised to get an expert opinion for the nasal findings. Flexible nasolaryngoscopy revealed hemorrhagic sinus drainage and fluid culture was collected from the right middle meatus. Karius’s panel was found to be negative. The fluid showed budding yeast on the gram stain and Micafungin was started. Point-of-Care Ultrasound (POCUS), a small amount of fluid occupying less than one rib space is observed at the base of the right lung. Figure 3 shows PET-CT of the skull base, indicating there is no significant uptake of FDG observed. The absence of significant FDG uptake in this image helps rule out malignancy and other potential causes of infection spread, focusing the diagnosis on localized sinusitis and bacteremia.

Autoimmune disorders were initially considered due to the positive titers of ANA. However, the rheumatologist ruled out autoimmune conditions based on several factors: the acute onset of symptoms following nasal surgery, the patient’s improvement with antibiotic therapy, and elevated WBC counts indicative of an infection rather than an autoimmune condition. Specific tests for autoimmune markers (e.g. antidouble-stranded DNA, anti-Smith antibodies) were negative, and there were no other systemic symptoms typically associated with autoimmune disorders, such as skin rashes, joint pains, or organ involvement. The patient’s clinical presentation and laboratory findings, including the response to antimicrobial therapy, supported the diagnosis of a nasal mucosal infection progressing to bacteremia, rather than an autoimmune etiology.

Empiric treatment with vancomycin and cefepime for presumed sepsis was initiated. Later, nasal wound culture showed multibacterial growths of Tsukamurella species, Enterococcus faecalis, Corynebacterium species, and Staphylococcus capitis. The culture was found to be sensitive to ciprofloxacin. Infectious disease specialist diagnosed the condition as nasal mucosal infection (sinusitis), which progressed to persistent bacteremia/ sepsis with arachnoiditis. The specialist recommended augmentin (875 mg amoxicillin component) twice daily orally, fluconazole (400 mg daily), and levofloxacin (500 mg daily). The patient improved and WBC counts returned to baseline. Two weeks after discharge, the patient reported recovery from illness.

## Discussion

Tsukamurella commonly presents as an opportunistic bacteremia causing pneumonia, cutaneous infection, meningitis, peritonitis, abscesses, and sepsis^7–9^. Tsukamurella is identified in immunocompromised individuals presenting as catheter-associated bacteremia. Tsukamurella pulmonis typically causes central line-associated bloodstream infections (CLABSI) from central venous catheters (CVCs) and Hickman catheters in patients with severe immunodeficiency or hematological malignancies. Rarely, T. pulmonis bacteremia can also occur with peripherally inserted central catheters (PICCs), highlighting its unpredictable nature^10^. It is also misdiagnosed as atypical mycobacteria presenting as pulmonary and ophthalmic infections^4,11–13^. Tsukamurella pneumonia can be easily misdiagnosed as pulmonary tuberculosis, especially in countries with a high tuberculosis burden, due to their similar clinical presentations. Tsukamurella should be carefully considered in the etiology of tuberculosis-like lung diseases^13^. T. pulmonis, referred to as an ‘ophthalmologic strain’, is speculated to possess adhesins for ocular surface cell binding and resistance to tear antibacterial substances^14^. Tsukamurella has also been reported to be associated with ANCA-associated small vessel vasculitis. Chronic Tsukamurella pulmonis infection can induce ANCA production and nephritis, closely mimicking ANCA-associated vasculitis. Accurate diagnosis is essential to avoid misdiagnosis and the potentially life-threatening consequences of inappropriate immunosuppressive therapy^11^.

In this patient, Tsukamurella presented as septicemia and frontal sinusitis in an immunocompetent individual, originating from mucosal infection.

Tsukamurella species present challenges for identification in clinical microbiology laboratories due to their phenotypic similarities with related genera and the limitations of traditional biochemical testing^1,2,15^. While initial differentiation can be achieved through gram staining, more reliable identification is achieved using DNA sequencing, PCR, and matrix-assisted desorption ionization-time of flight mass spectrometry (MALDI-TOF MS)^2,15,16^. Commonly, the patients are misdiagnosed, and later tissue sampling identifies Tsukumurella species. To identify Tsukamurella, molecular markers such as the sequencing of 16S rRNA, groEL, rpoB, secA1, and ssrA genes are used^16^.

Traditional biochemical tests often misidentify Tsukamurella because it resembles other corynebacteriales members, such as nocardia, rhodococcus, and gordonia. In contrast, DNA sequencing provides a more accurate identification by examining genetic markers that differentiate Tsukamurella from similar species. However, due to its complexity and expense, DNA sequencing is not commonly used in many clinical labs^17^.

MALDI-TOF MS is a cost-effective method for bacterial identification. It provides rapid and accurate species identification by analyzing bacterial protein profiles. When MALDI-TOF MS databases are updated with reference spectra for all Tsukamurella species, identification accuracy improves significantly. This highlights MALDI-TOF MS’s potential for routine use, as long as databases are regularly updated. Traditional methods, in contrast, often fail to identify Tsukamurella correctly due to its similarity to other genera^18^.

This patient was diagnosed with Tsukamurella infection after culturing the nasal wound. Levofloxacin was provided as therapy because the growths were multibacterial and were found to be sensitive to fluoroquinolones. Due to a lack of information and clinical experience, treatment guidelines have not been well established for Tsukamurella infections^3,19^. Tsukamurella is resistant to many penicillins, tetracyclines, and even third-generation cephalosporins^9,13^. Fourth-generation cephalosporins, fluoroquinolones, and imipenem have been effective for Tsukamurella^10,19–22^. Combination therapy can also be provided^2^. In this patient, levofloxacin and augmentin combination therapy has led to the resolution of the illness.

## Conclusion

In conclusion, Tsukamurella can involve mucosal tissues and present as septicemia unresponsive to Augmentin. The opportunistic nature does not limit the bacteria to immunocompromised hosts alone. Due to misdiagnosis, lack of timely intervention, accurate diagnostic tools, and treatment guidelines, Tsukamurella threatens hospitalized patients, underscoring the need for antibiotic susceptibility. In our case, combination therapy with fluoroquinolones and augmentin has shown to be effective.

## Ethical approval

This case report was conducted in accordance with the Declaration of Helsinki. The collection and evaluation of all protected patient health information was performed in a Health Insurance Portability and Accountability Act (HIPAA) – compliant manner.

## Consent

Written informed consent was obtained from the patient for publication and any accompanying images. A copy of the written consent is available for review by the Editor-in-Chief of this journal on request.

## Source of funding

The author(s) received no financial support for the research, authorship, and/or publication of this case report.

## Author contribution

All authors have equally contributed.

## Conflicts of interest disclosure

The authors declares no conflicts of interest.

## Research registration unique identifying number (UIN)

Not applicable.

## Guarantor

Not applicable.

## Data availability statement

Not applicable.

## Provenance and peer review

Not applicable.

## References

[1] Usuda D Tanaka R Suzuki M. Obligate Aerobic, gram-positive, weak acid-fast, nonmotile bacilli, Tsukamurella tyrosinosolvens: minireview of a rare opportunistic pathogen. World J Clin Cases 2022;10:8443–8449. 36157836 10.12998/wjcc.v10.i24.8443 PMC9453373

[2] Safaei S Fatahi-Bafghi M Pouresmaeil O. Role of Tsukamurella species in human infections: first literature review. New Microbes New Infect 2018;22:6–12. 29556401 10.1016/j.nmni.2017.10.002 PMC5857166

[3] Leung KCP Au SCL Ko TCS. Ophthalmic manifestation of Tsukamurella species: a case series and first report of ocular implant infection after enucleation. Cornea 2019;38:1328–1331. 31246677 10.1097/ICO.0000000000001997

[4] Park BJ Goosey JD Belloso M. Tsukamurella keratitis: the first case in the United States. Can J Ophthalmol 2021;56:e153–e155. 33839066 10.1016/j.jcjo.2021.03.005

[5] Tam PMK Young AL Cheng L. Tsukamurella: an unrecognized mimic of atypical mycobacterial keratitis? The first case report. Cornea 2010;29:362–364. 20098312 10.1097/ICO.0b013e3181ae2594

[6] Riley DS Barber MS Kienle GS. CARE guidelines for case reports: explanation and elaboration document. J Clin Epidemiol 2017;S0895-4356:30037–30039. 10.1016/j.jclinepi.2017.04.026 28529185

[7] Kawashima A Kutsuna S Shimomura A. Catheter-related bloodstream infection caused by Tsukamurella ocularis: a case report. J Infect Chemother 2022;28:434–436. 34802889 10.1016/j.jiac.2021.11.003

[8] Kuge T Fukushima K Matsumoto Y. Chronic pulmonary disease caused by Tsukamurella toyonakaense. Emerg Infect Dis 2022;28:1437–1441. 35731181 10.3201/eid2807.212320 PMC9239891

[9] Malik Z Shah PJ Tariq F. First report of Tsukamurella endocarditis in an immunocompromised patient receiving chemotherapy. Eur J Clin Microbiol Infect Dis 2020;39:1989–1991. 32361958 10.1007/s10096-020-03917-5

[10] Suzuki J Sasahara T Toshima M. Peripherally inserted central catheter-related bloodstream infection due to tsukamurella pulmonis: a case report and literature review. BMC Infect Dis 2017;17:677. 29020942 10.1186/s12879-017-2796-8 PMC5637316

[11] Ochi K Mukai T Ota S. Tsukamurella pulmonis central venous catheter infection mimicking proteinase 3-antineutrophil cytoplasmic antibody (PR3-ANCA)-associated vasculitis. Immunol Med 2021;44:211–215. 32649848 10.1080/25785826.2020.1791403

[12] Chen C-H Lee C-T Chang T-C. Tsukamurella tyrosinosolvens bacteremia with coinfection of mycobacterium bovis pneumonia: case report and literature review. Springerplus 2016;5:2033. 27995010 10.1186/s40064-016-3707-y PMC5128003

[13] Yang L Cao Y Dan Z. Community-acquired Tsukamurella pneumonia in a young immunocompetent adult: a case misdiagnosed as pulmonary tuberculosis and literature review. Postgrad Med 2017;129:563–566. 28628338 10.1080/00325481.2017.1344513

[14] Woo PC Fong AH Ngan AH. First report of Tsukamurella keratitis: association between T. tyrosinosolvens and T. pulmonis and ophthalmologic infections. J Clin Microbiol 2009;47:1953–1956. 19369436 10.1128/JCM.00424-09 PMC2691093

[15] Sheng W-H Huang Y-T Chang S-C. Brain abscess caused by Tsukamurella tyrosinosolvens in an immunocompetent patient. J Clin Microbiol 2009;47:1602–1604. 19297591 10.1128/JCM.01932-08 PMC2681870

[16] Hsueh PR Lee TF Du SH. Bruker biotyper matrix-assisted laser desorption ionization–time of flight mass spectrometry system for identification of Nocardia, Rhodococcus, Kocuria, Gordonia, Tsukamurella, and Listeria Species. J Clin Microbiol 2020;52:2371–2379. 10.1128/JCM.00456-14 PMC4097692 24759706

[17] Teng JL Tang Y Chiu TH. The groEL gene is a promising target for species-level identification of Tsukamurella. J Clin Microbiol 2017;55:649–653. 27974536 10.1128/JCM.02260-16 PMC5277538

[18] Teng JLL Tang Y Wong SSY. MALDI-TOF MS for identification of Tsukamurella species: Tsukamurella tyrosinosolvens as the predominant species associated with ocular infections. Emerg Microbes Infect 2018;7:80. 29739926 10.1038/s41426-018-0083-4 PMC5940693

[19] Perez VA de J Swigris J Ruoss SJ. Coexistence of primary adenocarcinoma of the lung and Tsukamurella infection: a case report and review of the literature. J Med Case Rep 2008;2:207. 18554413 10.1186/1752-1947-2-207 PMC2442117

[20] Schwartz MA Tabet SR Collier AC. Central venous catheter–related bacteremia due to tsukamurella species in the immunocompromised host: a case series and review of the literature. Clin Infect Dis 2002;35:e72–e77. 12228839 10.1086/342561

[21] Bouza E Pérez-Parra A Rosal M. Tsukamurella: a cause of catheter-related bloodstream infections. Eur J Clin Microbiol Infect Dis 2009;28:203–210. 18810513 10.1007/s10096-008-0607-2

[22] Romano L Spanu T Calista F. Tsukamurella tyrosinosolvens and rhizobium radiobacter sepsis presenting with septic pulmonary emboli. Clin Microbiol Infect 2011;17:1049–1052. 20946410 10.1111/j.1469-0691.2010.03396.x

